# Developing a cardio-oncology education programme for nurses

**DOI:** 10.1016/j.apjon.2026.100934

**Published:** 2026-03-11

**Authors:** Gerry Lee, Lisa Nodzon, Chelsea P. Kriesberg, Asma Al Bulushi, Priya Reehal, Edith Pituskin, Anecita Fadol

**Affiliations:** School of Nursing & Midwifery, Brookfield Health Sciences Complex, University College Cork, Cork, Ireland; Advanced Registered Nurse Practitioner, Department of Malignant Haematology, H. Lee Moffitt Cancer Centre & Research Institute, Tampa, FL, USA; Nurse Practitioner, Pediatric Cardiology and Pediatric Cardio Oncology, Advocate Children's Hospital, Oak Lawn, IL, USA; Clinical Nurse Specialist, Nursing Administration, National Center for Cancer Care and Research, Hamad Medical Corporation, Doha, Qatar; Cardio-Oncology Centre of Excellence, Royal Brompton Hospital, London, United Kingdom; Mary Stuart, Nurse Practitioner AYA Program, IWK Health Centre, University Avenue, Halifax, Canada; Professor, Faculty of Nursing, University of Alberta, Alberta, Canada; Associate Professor, Departments of Nursing & Cardiology, UT MD Anderson Cancer Center, Houston, TX, USA

**Keywords:** Cardio-oncology, Nursing education

As populations age, the likelihood of living with both cancer and cardiovascular disease (CVD) has become more commonplace. While advances in treatment have transformed outcomes for both conditions, their coexistence presents a complex set of issues. For many patients, cancer treatment begins with a history of established cardiovascular risk factors such as hypertension or hypercholesterolaemia. For others, cardiovascular complications including heart failure, acute coronary syndrome, or atrial fibrillation emerge during therapy as a consequence of cancer treatments (known as cardiotoxicity). When these cardiovascular complications occur, the boundaries between the specialties blur, and care must be thoughtfully coordinated between oncology and cardiology.

Cardio-oncology has emerged in response to this evolving need. As a developing discipline, it represents more than a collaboration between two specialties; it reflects a growing recognition that patient care spans multiple specialties. As such, patient care demands an integrated approach that acknowledges the physiological and psychological challenges that accompany dual diagnoses.

Within this landscape, the role of the cardio-oncology nurse is pivotal in bringing together oncology and cardiology expertise within cohesive, interdisciplinary teams via a coordinated approach. The cardio-oncology nurse addresses patients’ multifaceted needs throughout the cancer pathway. Faced with the risk of cardiotoxicity during and after cancer treatment, nurses are often the first to recognise signs of cardiotoxicity by assessing, monitoring, and managing patients. The cardio-oncology nurse also provides continuity following successful cancer treatment, ongoing patient education and reassurance and helping patients navigating their health.

In 2024, the International Cardio-Oncology Society (ICOS) nurses working group undertook a needs assessment of nurses. The results from 305 respondents confirmed the majority had no formal education in cardio-oncology (*n* = 268, 88%). Four-fifths (247/308, 80%) had a strong desire to learn more about cardio-oncology and were interested in formal learning programs that would lead to certification.^1^ The ICOS nurses working group met and agreed that an education programme was needed. A planning committee was established and subsequently identified topics for a cardio-oncology programme to ensure it reflected all aspects of cardio-oncology care. Experts in the cardio-oncology field were identified and invited to develop a module. These experts including nurses and physicians were from the USA, UK, Canada, Ireland, China and Qatar. Once agreed, each expert recorded a 30 or 60-min presentation and submitted several questions for the pre/post-test assessment. The finalised programme, Cardio-oncology Nursing Education (CONE) programme, in collaboration with MD Anderson Cancer Centre, was launched in November 2025 and is a certified programme (ANCC 18.25 nursing contact hours and 10.25 hours in Pharmacotherapeutics).^2^ Each module has pre and post test questions and users need to pass each module by gaining 70% or more in each completed module.

This initiative seeks to deepen nurses' understanding of the complex relationship between cancer and CVD, enhance the early identification and management of treatment-related cardiovascular toxicities, and guide the development of comprehensive cardio-oncology services. At its core, it underscores the importance of integrating supportive care and proactive cardiovascular risk management into the broader cancer care continuum. The CONE programme is available online and nurses can undertake modules in their own time. There are a total of 27 modules and covers Cardiovascular Assessment, The Role of Electrocardiography and diagnostic tests and covers all the relevant medications across the different types of cancers, for example there are modules on Bruton's Tyrosine Kinase Inhibitor and Proteosome inhibitors. The modules provide an overview of various cancers as well as how to manage cardiotoxicities. The psycho-social aspects are also included and there are modules on survivorship, patient and family education, the social determinants of health and end-of-life care. From an organisational perspective, there are modules on developing a cardio-oncology service.

The aim is to evaluate the programme in 2027 via an online survey. The survey would include demographic data on CONE participants (current role, location, etc) and questions gaining their opinions on the value and applicability of the modules to their practice and how the modules impacted on their practice. There will be an option for free-text comments with reference to whether quality indicators have improved in their practice: examples include early detection of cardiotoxicity, improved treatment times and increased uptake of multidisciplinary referrals. Thus, changes in clinical practice will be explored via the survey and potential impact on patient-related outcomes.

In summary, cardio-oncology has emerged at the intersection of two of the most significant health challenges of our time: cancer and CVD. As cancer therapies advance and survivorship improves, the unintended cardiovascular consequences of treatment have become increasingly visible. This growing burden of cardiotoxicity has created a global imperative for more integrated, specialised models of care and we hope that the CONE programme provides a worthy resource in this evolving new specialty.

## Declaration of generative AI and AI-assisted technologies in the writing process

No AI tools/services were used during the preparation of this work.

## Declaration of competing interest

The authors were involved in the development of the CONE programme. They received honoraria for their contributions to the programme development and educational content. The authors declare no other financial or commercial relationships related to this work.
